# New technologies and related changes at work as triggers for professional development in the nursing domain: an exploratory interview study

**DOI:** 10.1186/s12912-025-03291-7

**Published:** 2025-06-17

**Authors:** L. Romina Bornhaupt, Regina H. Mulder

**Affiliations:** Faculty of Human Sciences, UR - University of Regensburg, Universitätsstraße 31, 93053 Regensburg, Germany

**Keywords:** Healthcare, Nursing, Professional development, Technology, Change at work, Learning, Workplace learning, Interview study, Critical incident technique

## Abstract

**Background:**

Technological developments are changing work in healthcare. To keep up with these changes, nurses need to continuously adapt which requires three components of professional development: Elaboration, expansion, and externalization. Few studies, with partly inconsistent results, address technology-driven changes at work in the nursing sector. However, the implications of these changes for nurses’ professional development remain unclear. The aim of the present study was therefore to explore the changes at work resulting from the implementation of technology, which learning activities emanate from these changes, and what the triggers are for professional development.

**Method:**

An exploratory, qualitative research design was applied. Data collection was conducted through semi-structured interviews using the Critical Incidents Technique. Ten nurses with state-approved professional qualification in nursing, geriatric care, or equivalent nursing training took part in the study. Data analysis was conducted in a combination of deductive and inductive qualitative content analysis using a concept-driven coding frame.

**Results:**

Changes in work characteristics were reported for each category of technology. Regarding the individual categories of work characteristics (task, social, contextual), however, partly different changes were mentioned in relation to the technology categories. Learning activities from all three categories were identified. There are indications that both the usage of technology itself as well as the consequential changes at work can trigger professional development. However, differences were reported in terms of the components of professional development: Mentioned was that elaboration was triggered by the usage of technology and changes at work, and that expansion was related to problems and knowledge gaps associated with the technology and related changes. Externalization, in turn, was linked to social aspects and staff responsibility.

**Conclusions:**

The results show how nurses can learn due to technology-driven changes at work. Furthermore, initial insights were gained on how work characteristics, learning activities and triggers are related. These findings can help to consider the specifics of the situation when planning the implementation of technologies, processes that results from that, and how professional development can be fostered.

**Supplementary Information:**

The online version contains supplementary material available at 10.1186/s12912-025-03291-7.

## Background

Technological developments are changing work in healthcare [1, 2]. To keep up with these changes, professionals need to continuously adapt and (further) develop their competences. This improvement of professionals’ competences needed due to changing requirements of today’s world of work is called professional development and consists of three different components of work-related learning [3]: (1) *Elaboration* refers to all activities that lead to further development of one’s own competences by using one’s own experiences for learning from and during work. Learning takes place, for example, by reflecting on one’s own behaviour or on mistakes. This form of learning can be implicit. (2) *Expansion* refers to all activities that lead to further development of one’s own theoretical and conceptual understanding through confrontation with external input. This input can originate from printed or electronic media as well as in various forms of social interaction, such as discussions or feedback. (3) *Externalization* leads to further development of one’s own competences as well as the development of the team and/or the organization through the explication of one’s own knowledge, such as sharing or codifying knowledge.

These three components of professional development are induced by technology-driven changes in the workplace, which can lead to a disruption of routines so that existing knowledge, skills, and practices are no longer sufficient [e.g., 4, 5]. Understanding how technology can change work in healthcare, requires an understanding of what characterizes work. Morgeson and Humphrey [6] distinguish three categories of work characteristics:

(1) *Task characteristics* include all aspects that relate to the way in which work itself is accomplished or that emerge from the actual work tasks. This contains, for example, autonomy as well as task content. It also includes the demands on individual knowledge, skills and abilities created by the tasks, such as cognitive and emotional demands, as well as complexity. For example, the implementation of various health information technology solutions can lead to an increase in time required for documentation [7], an increase in standardisation of work tasks, and a decrease in autonomy [8]. Similarly, the implementation of a barcode administration system for medication can challenge the autonomy of nursing staff due to its rigidity [9]. According to Fachinger and Mähs [1] the implementation of electronic documentation systems can lead to time savings, for example, using text modules or the automated recording of data by monitoring systems, and the time gained can be invested in nursing tasks. At the same time, more time must be spent in the implementation and changeover phase to learn how to use the technology and, if necessary, to adapt routine processes to new structures.

(2) *Social characteristics* refers to the social environment in which work is performed, such as social support and social interaction. For example, the usage of IT technologies can make collaboration with colleagues more accessible [10], or an instant messaging application for smartphones can promote the relationship between caregiver and patient in home care, as the system helps to show difficult-to-express emotions as well as by reducing language barriers [11]. Another example is the decrease in social interaction through the usage of an electronic documentation system in outpatient care, as the usage of the system can eliminate the need for personal handovers [12].

(3) *Contextual characteristics* include all aspects that are not directly related to a single task, but to the broader context (physical and organizational) in which tasks must be performed and which arise from the work environment, such as physical demands and equipment use [6]. An example is that the usage of exoskeletons reduces physical demands during care tasks [13]. In terms of clinical technology and information technology, its implementation can lead to an increased workload (in the sense of having to perform additional tasks) due to higher efficiency and patient flow rates [14]. However, empirical evidence on technology-driven changes at work in nursing is - according to the technology under consideration - currently scarce and partly inconsistent [1].

The term technology encompasses a broad variety of systems, devices or aids, and universal definitions are lacking [15]. In general, technology can be defined as digital or mechanical tools, devices or systems that replace or complement the accomplishment of work tasks [16, 17]. Work tasks may require complex systems with technological highly diverse functionalities. In addition, new technologies are becoming more and more autonomous, eliminating the need for manual operation [17]. Therefore, a distinction according to the functionality of the technology and its interaction with humans seems useful. Three categories of technology can be distinguished [12]: (1) *Communication supporting technologies* require the manual involvement of at least two people and connect them functionally in a synchronous or asynchronous manner. For instance, electronic documentation system can enable both the pure exchange of information as well as interaction between people. (2) *Task-supporting technologies* require the intentional operation of at least one human being since the interaction between human and device forms the basis for its functionality. Through this interaction, for instance using patient lifts, the manual process of executing work tasks is supported. (3) *Task-replacing technologies* replace, in whole or in part, the process of performing a work task by itself or by interacting with other technologies, without requiring permanent human involvement. Task-replacing technologies, such as hybrid closed-loop insulin deliver systems, require programmed instructions and rules to provide the control and execution of repetitive and consistent processes.

**Fig. 1:**
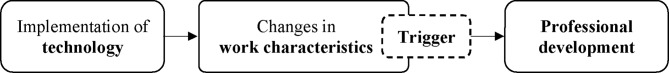
Conceptual model

However, the constructs considered so far should not thought of in isolation. Figure 1 shows the relationships between the constructs. Based on the distinction according to the degree of human interaction, it is conceivable that the implementation of technologies from different categories may lead to different changes at work. These changes induce cognitive conflicts [18, 19] which can be resolved through engaging in learning activities [20]. Professional development is the engagement in learning activities. Hence, the changes in work characteristics are used as triggers for professional development [21].

The available studies in nursing that deal with the isolated constructs so far lack an integrative view that represents these constructs in their context, more specifically the links between the constructs. The aim of this study is to gain an initial insight into the relationships between new technology, changes at work, triggers and professional development. Therefore, the following research questions will be answered using an explorative approach:

1. How does the implementation of new technology change work in nursing?
2. Which learning activities arise from the implementation of technology and related changes at work?
3. What triggers these learning activities?

The results obtained from this study will serve as a foundation for formulating hypotheses. These hypotheses will enable empirical testing to derive implications for practice. This ensures valid and reliable findings for deriving recommendations for change processes in the context of technology implementation. Additionally, the gained insights can be used to raise nurses’ awareness of their own professional development. Managing their own professional development is critical for maintaining professional performance and adapting to change. Furthermore, the findings help leaders understand the development of their team members and reflect on change processes within their organisation by for instance implementing new technology. By understanding how their team members develop, leaders can provide targeted support and create a supportive environment.

## Method

### Study design and data collection

To investigate how the implementation of technology and associated changes relate to professional development, an explorative interview study was conducted. Therefore, qualitative data was collected through semi-structured interviews. To stimulate participants’ narratives, an interview guide (see Additional file 1) was developed for this study using the Critical Incidents Technique (CIT) [22, 23]. The CIT is an effective method to assess human experiences and behaviours [24]. Flanagan [22] describes incidents as “any observable human activity that is sufficiently complete in itself to permit inferences and predictions to be made about the person performing the act” (p. 327). Since critical incidents are defined in relation to the study objective [24], the nurses’ professional development caused by the implementation of a new technology and related changes was investigated. At the beginning of each interview, participants were asked to recall the following situation: *Please think about the last time a new technology was introduced as part of your work and describe that situation*. To obtain detailed and rich descriptions of the situation and participants’ behaviours, the guide included open-ended follow-up questions about (1) the technology addressed, (2) the resulting changes at work (research question 1), (3) the learning activities that followed (research question 2), as well as (4) their specific triggers (research question 3).

All interviews were conducted and recorded via video conferencing software. Written informed consent for data collection and recording was obtained before each interview. Interviews lasted between 38 and 133 min (*M* = 67.32; *SD* = 26.13). After each interview, participants were sent an additional demographic data questionnaire. The recorded interviews were transcribed verbatim and in anonymized form.

### Participants

To obtain broad insight into the current state of new technologies in nursing and their consequences, various selection criteria were taken into account: Firstly, participants had to hold a state-approved professional qualification in nursing, geriatric care, or equivalent nursing training. The study’s target group was deliberately defined broadly. Until 2020 there were specific studies for general nurses, geriatric nurses and paediatric nurses in Germany (level 4 of the European Qualifications Framework, EQF). They each lasted three years. As recently started nurses have little work experience and therefore limited opportunities for comparing situations (such as before and after the implementation of new technologies), only nurses with longer professional experience (> 5 years) were included in the study. Furthermore, nursing staff from both inpatient and outpatient care were considered. There were no restrictions in terms of medical specialties during the entire acquisition process. Initially, fifteen regional hospitals, care facilities and retirement homes were contacted. Due to a low response rate, the number of participants obtained in this manner was expanded through the authors’ professional network and additional snowball sampling. The sample gained in this way covers a wide variety of the target group. Pilot projects and thus technologies that do not correspond to current practice were not considered.

A total of 10 registered nurses (seven females and three males) participated in the study (Table 1). Together they covered technologies and learning activities of all categories. Participants’ ages ranged from 26 to 60 years (*M* = 38.20; *SD* = 11.61), and their working experience, including training time, ranged from 6 to 26 years (*M* = 12.90; *SD* = 6.33).

**Table 1:** Characteristics of participants

*P*	Care setting	Position	Experience (years)^1^
1	Inpatient mental health care	w/o staff responsibility	6
2	Intensive care unit care	Practical trainer	13
3	Outpatient intensive care	Care management	10
4	Residential long-term care	Care management	21
5	Outpatient intensive care	Care management	18
6	Residential long-term care	Practical trainer	11
7	Intensive care unit care	w/o staff responsibility	9
8	Intensive care unit care	w/o staff responsibility	9
9	Residential long-term care	Care management	6
10	Intensive care unit care	w/o staff responsibility	26

Note. P = participant; w/o = without; ^1^work experience including training time in years

### Data analysis

Data was analysed using a combination of deductive and inductive qualitative content analysis [25]. Therefore, the analysis was performed in several steps: First, a concept-driven coding frame was developed. The four main categories were selected based on key variables in the research questions, namely the technology, the related changes in work characteristics, the professional development that emanates from that and its specific triggers. They are therefore congruent with the categories covered by the questions in the interview guide. Suitable constructs for answering the research questions were selected based on the analysis of literature. The definitions of all categories and subcategories used were derived deductively from these constructs and their definitions. Initial examples for codes were also derived from these sources. They were complemented by codes that were inductively derived from the material during the test coding and the main analysis. To answer the research questions, the coding frame included the following main categories, as well as their subcategories, first codes derived from theory, and definitions: (1) technology [12], (2) work related changes [6, 26], (3) professional development [3, 27], and (4) triggers [4, 28]. The developed coding frame was checked by two colleagues for its comprehensibility, completeness, and usability. One of the two researchers is investigating the same topic in a different domain, and the other has experience in qualitative content analysis. An overview of the main categories, subcategories and exemplary codes used in the analysis is presented in Table 2.

**Table 2:** Overview of the main categories, subcategories, and exemplary codes

Category	Subcategory	Examples of codes
Technology	Communication supporting technology	Electronic documentation system* Information management system*
	Task-supporting technology	Apps and tablets for entertainment* Label printer for infusions*
	Task-replacing technology	Chest compression system* Ventilator*
Work related changes	Task characteristics	Autonomy Degree of formalization*
	Social characteristics	Responsibility* Social interaction
	Contextual characteristics	Physical demands Traceability*
Professional development	Elaboration	Experimenting Testing something in a safe space*
	Expansion	Looking up information Receiving advice from experts*
	Externalization	Convincing others* Sharing information
Trigger		Problem Colleague behaviour*

Note. * Examples for data-driven codes

To check the suitability of the coding frame a trial coding was carried out. Special attention was paid to whether the definitions of the codes allowed a clear assignment. Therefore, three transcripts were first segmented into meaningful units using a thematic criterion [25]. Subsequently, these segments were first assigned by one author to the main categories and subcategories and finally the specific codes using MAXQDA 12. As it was not clear before the start of the study which technologies would be mentioned, all codes were derived inductively from the individual interview material. The subcategories of this category (*communication supporting*, *task-supporting*, & *task-replacing technology*) proved to be suitable for the technologies mentioned. Furthermore, the test coding showed that codes in the professional development category were easy to apply to the data material. In this case, only individual codes were inductively derived from the data material. This was particularly necessary for the codes in the externalisation category, as many new learning activities were found in the data that did not appear explicitly in the literature (e.g., *convincing others*). For the category *changes in work characteristics*, on the other hand, codes mainly had to be derived from the data material. These were presented together with anchor examples and definitions to the researcher familiar with the research field and their plausibility was discussed and revised if needed. Examples are the codes *degree of formalization* (task characteristics), *responsibility* (social characteristics), and *traceability* (contextual characteristics). One example of an adapted definition is the code *complexity*. Due to the ambiguity of the term complexity and its various definitions, this study finally referred to the definition by Wood [29]. The revised coding frame was then used for the main analysis phase. The main analysis was closely related to the test coding procedure. In the first step, the material was divided into coding units. These units could consist of individual words or short sections of text and were based on a thematic criterion. This means that text passages were identified as a unit if they had a thematic relevance to one of the main categories [25]. In the next step, these units were assigned to the subcategories/codes. If there was not yet a suitable code, the section was marked separately, and a working title was chosen for the code. During further analysis these working titles were adapted if necessary or adopted if they proved to be suitable. All codes derived during this phase were discussed with the second researcher before being finally integrated into the coding frame. Table 3 shows three examples of coding with categories, codes, and the corresponding text passages.

**Table 3:** Coding examples for each main category

Meaning unit	Code	Subcategories	Category
“The last change we had on the ward was the implementation of an electronic patient chart. So our complete documentation system and patient monitoring.”	Electronic documentation system	Communication supporting technology	*Technology*
“The documentation of individual steps then seems more difficult to me, or suddenly needs more clicks, where I otherwise only had to write one sentence.”	Complexity (increase)	Task characteristics	*Changes in work characteristics*
“I also think a lot for my colleagues: How do I make it more manageable for my older colleagues? And when it’s clear to me, but others say I’m struggling, then I think: Why? What is the difference between us? What could be easier?”	Reflecting	Elaboration	*Professional Development*
	Colleague behaviour		*Trigger*

To ensure the quality of data coding, a total of three randomly selected interviews were coded as entity by two different researchers. Since the second coder is working on the same research topic but in a different domain, and due to the specificity of the domain, coder 1 first segmented the transcript into conceptually meaningful units, which were then coded independently by both [30, 31]. Interrater reliability for the three interviews (Cohen´s κ = 0.730; *p* < .001) indicates a substantial agreement [32].

## Results

Technologies from each category were addressed during the interviews. However, electronic documentation systems (*communication supporting*) were mostly mentioned [P1, P2, P3, P5, P6, P7, P9]. Also assigned to this category were information management systems on the intranet with multimedia content [P1]. Two technologies could be categorized as *task-supporting*: Apps and tablets to provide entertainment for care recipients [P4], and label printer for infusions [P8]. Four *task-replacing* technologies were mentioned: Automated cuff pressure controllers [P8], continuous glucose monitor systems with sensor, reader, and computer app [P4], chest compression systems [P7], and ventilators [P3, P10]. Below, the codes derived inductively from the data material are marked with an asterisk (*).

### Changes in work characteristics due to the implementation of new technologies

#### Changes caused by communication supporting technologies

Changes at all categories of work characteristics were described for communication supporting technologies (see Table 4). Regarding task characteristics, the automation of task steps* (e.g., through interfaces between electronic documentation system and monitoring), as well as an increase in *complexity* (due to an increase in the number of distinct acts needed to perform the task) and an increase in the degree of formalization* (e.g., by setting a maximum number of characters) were reported as a result of the implementation of electronic documentation systems. Furthermore, a decrease in *autonomy* in the execution of tasks due to defaults of the system was mentioned. In this case, an automatically activated alarm signal forced the nurse to take action when vital signs were entered that did not correspond to the guideline values:

“I don’t have an alarm signal there [in the handwritten documentation]. I have this target value, I have the alarm limits, and then I have to use my nursing expertise and consider independently: What are the consequences? Do I do something here, do I do nothing here? And with the digital version, I have to react. Because otherwise I have the red triangle in it all day.” [P5].

**Table 4:** Changes in work characteristics listed by technology category

Work characteristic	Coding	Communication supporting	Task-supporting	Task-replacing
Task	Automation of task steps*	+	+	+
	Autonomy	−		
	Cognitive demands	−	−	− +
	Emotional demands			− +
	Complexity	+		−
	Degree of formalization*	+		
	Task content*	×		×
	Time required for the task*	− +	−	−
Social	Responsibility*	−		
	Social interaction	−		
Contextual	Physical demands			−
	Temporal-spatial flexibility*	− +		
	Traceability*	+		
	Workload*	+		

Note. – Decrease, + Increase, × Change in content, *inductively derived code

A decrease in *cognitive demands* (due to for instance text modules or simplified forms of presentation) and in the time required for the task* (e.g., because of automation or simplified presentation) were mentioned for both the electronic documentation systems and the information management system. The examination of an individual case shows that the simultaneous increase and decrease of certain work characteristics has been mentioned and, in addition, preceding changes (here the increase in *complexity* and the automation of task steps*) resulted in these contradictory changes:

“So, on the one hand, the documentation has been extended in my opinion. Because it’s very complicated and involves a lot of clicks to enter the simplest things, it often takes me longer. This minimal part, where I don’t have to enter any more values, has saved me a bit.” [P2].

Moreover, for communication supporting technologies a change in task content* was reported. Specifically, because of saving time on documentation, the participant was able to use the time freed up for care activities:

“For me it’s nice because it’s just takes less time at that point. Because I say, okay you can just concentrate more on your core business, which is the care.” [P3].

In terms of social characteristics, a decrease in responsibilities* was mentioned. In this specific case, the implementation of an electronic documentation system led to the documentation no longer being signed off by the nursing manager (as in the paper version) but by assistants and nursing students themselves. This meant that they were also directly held responsible for any errors in the documentation. In another case, a decrease was reported in *social interactions* because of the decrease in temporal-spatial flexibility*:

“Actually, it was always the case that we talked to each other again for the last half hour before the next shift. No matter what it was about. It wasn’t even work-related. It was basically psycho-hygiene or just having a cigarette. […] And that sometimes falls apart now because colleagues often end up documenting.” [P2].

Regarding contextual characteristics, only changes related to electronic documentation systems were mentioned. These were an increase in traceability*, as well as an increase in workload* because of the delegation of additional new tasks (due to time savings by using the technology). Both increases and decreases in temporal-spatial flexibility* due to the usage of the technology were reported. These contrasting changes were described by different participants, as they had different hardware available (tablets vs. computers in the patient’s room) and one person repeatedly experienced internet malfunctions, making it only possible to work with the system at certain times.

#### Changes caused by task-supporting technologies

The only changes in work characteristics regarding task-supporting technologies mentioned were the automation of task steps* and, therefore, a decrease in *cognitive demands* and a decrease in the time required for the task* were described in relation to label printer for infusions.

#### Changes caused by task-replacing technologies

For task-replacing technologies, only changes in task and context characteristics were described. Changes in task characteristics were the automation of task steps* (described in relation to every technology in this category) and, in consequence, a decrease in *complexity* (described in relation to automated cuff pressure controller and continuous glucose monitor systems) and a decrease in *cognitive demands* (described in relation to continuous glucose monitor systems and ventilators). At the same time an increase in *cognitive demands* due to the usage of a new ventilator, especially during the changeover process, was mentioned:

“And then you’re standing there, and you have to quickly switch from one ventilation mode to another and suddenly you have to swipe instead of press. So that is really cognitively challenging.” [P10].

Similarly, both an increase and a decrease in *emotional demands* due to the usage of chest compression systems and new ventilators were described. In the case of ventilators, this change was both times due to insecurity in the usage of the devices. In one case, this uncertainty was reduced by more automation of the device, while in the other case it was increased by an unfamiliar user interface:

“Of course, that causes stress. Enormous stress because the patients were incredibly vulnerable, there could be an intubation situation at any time and then I’m standing there, and I have a device that I’m not familiar with.” [P10].

In relation to the chest compression system, the opposite changes in *emotional demands* were reported again by the same participant:

“It’s not very professional, but somehow, you’re still emotionally involved. […] And [the chest compression system] takes that away from you a little bit. That you at least have this physical distance to it. You also don’t feel this physical exhaustion, which in turn might lead to emotional exhaustion or something. On the other hand, I also have to say that seeing [the chest compression system] in action is not a pretty sight. […] It looks very upsetting, very dehumanized anyway.” [P7].

In addition, a decrease in time required for the task* due to the automation of task steps* by continuous glucose monitor systems and ventilators was mentioned. One change regarding task-replacing technology was again related to task content*. By automating the task, it was possible to take on more challenging tasks in the resuscitation process:

“That means that the simplest activity or the least challenging activity is taken off my hands. And that actually improves my job profile because it gives me the opportunity to perform higher-level tasks. To perform more important, more challenging tasks that I wouldn’t be able to do otherwise, because I would have to do chest compressions.” [P7].

The only change in contextual characteristics was identified related to chest compression systems. Here, there was a decrease in *physical demands* due to the usage of the system. Table 4 provides an overview of the results with respect to technology-related changes at work for the three categories.

### Learning activities and their triggers arising from the implementation of new technologies

#### Elaboration

In relation to learning activities, examples in each category were identified in the data. *Experimenting* related to electronic documentation systems and ventilators was mentioned. This involved in one case familiarizing oneself with the functions of the device. In another case, a geriatric nurse first had to experiment how to integrate the electronic documentation device into her workflow:

“Well, I’ve also tried a lot of things. At the beginning, I still clamped the tablet under my arm, but that didn’t work at all, because it either fell or someone [a resident] stole it. […] And now, I always use the nursing trolley and simply realize that I can leave it on it. I know the resident can’t get to it directly, can’t read anything, can’t accidentally turn something off or click on something.” [P9].

Experimenting was mainly triggered by the usage of the technology itself, but in one instance by a problem. In this case, the system’s defaults for medication ordering were not appropriate due to an increase in the degree of formalization, and a solution had to be found to outsmart the system. Also described in the interviews were testing something in a safe space* in the context of electronic documentation systems. In this instance, it was described that a test version could be used to try out the entry and saving of data without actually entering it into the system. In relation to communication supporting and task-replacing technologies, *learning by doing* was further mentioned. In all cases, the latter two forms of learning activities were triggered by the usage of the technology itself. Moreover, a learning activity assigned to the elaboration category was *reflecting*. This learning activity was the only one in this category that was triggered by a change in work characteristics, namely the decrease in temporal-spatial flexibility and the question of whether this fits in with one’s own way of working as well as the increase in complexity and related colleague behaviour:

“I also think a lot for my colleagues: How do I make it more manageable for my older colleagues? And when it’s clear to me, but others say I’m struggling, then I think: Why? What is the difference between us? What could be easier?” [P2].

Even though the colleagues were considered, this quote was coded as elaboration, as this reflection led to the acquirement of solely her own knowledge. Subsequently, through a next learning activity, the knowledge gained was used to help colleagues and shared (externalization).

#### Expansion

Different expansion learning activities could be identified. *Asking others* was reported frequently. The source of information in relation to an automated cuff pressure controller, for example, were colleagues. Here, the learning activity was triggered by the need to use the technology itself:

“I think it was the colleague from whom I took over the shift. I think that I just noticed it [the device] and that I simply asked. And then she explained it to me briefly.” [P8].

Asking colleagues was triggered by problems in working with the technologies (electronic documentation systems and ventilators). In one case, the problem was due to a change in task characteristics, namely an increase in the degree of formalisation. In addition, experts (such as IT specialists) and/or superiors were asked in relation to electronic documentation systems and continuous glucose monitor systems. These learning activities were also triggered by problems. In some cases, there were also specially trained multipliers within the organisation to whom questions were directed. These learning activities were triggered both by the usage of the electronic documentation system itself and by problems related to it - again caused by a change in task characteristics (increased degree of formalization). Regarding electronic documentation systems and ventilators, *looking up information* in internal or external sources (e.g., information management system, manual, standard operating procedure, internet) was reported. This was triggered in some cases by problems in using the technology and in one case by a gap in knowledge, revealed by performing unusual or new tasks (related to the technology):

“I wanted to print a nursing report and I didn’t know how to do that. None of the multipliers knew either, so I sat down and looked at the online manual until I figured it out.” [P6].

Almost all participants stated that they had taken part in some form of *formal training* (related to electronic documentation systems, continuous glucose monitor systems, and ventilators). These trainings mostly took place before or at the beginning of the implementation process and varied greatly in terms of duration and methodological design. Regarding ventilators, reading the manual* without having a specific question was mentioned. In the context of electronic documentation systems, receiving advice from experts* and receiving briefings from colleagues* were also described. All three learning activities were triggered by the usage of the technology. Finally, *receiving feedback* and receiving guidance from colleagues* were reported. Both were related to a chest compression system and were triggered by a knowledge gap related to a new task:

“If I were alone during a resuscitation, I wouldn’t dare to use the device. I need my colleagues or especially those from the emergency room to help me, to support me. Because I don’t have the knowledge, or at least not to a sufficient degree. […] I have already installed the device myself, but only with guidance.” [P7].

#### Externalization

The externalisation learning activities were related to communication supporting technologies. One learning activity related to electronic documentation systems was addressing common mistakes in team meetings*. In this case, the learning activity of a person in a leadership position was triggered by the observation that employees repeatedly made the same mistakes:

“And every now and then, when you notice, okay, some source of error is just too high or several employees are making the same mistakes, we simply address that in team meetings.” [P3].

In addition, in relation to electronic documentation systems, *giving feedback* was reported. On the one hand, this was triggered by a change in social characteristics, namely the decrease in responsibility while at the same time transferring it to the employees and the goal of the superior that they do not feel left alone with this. On the other hand, it was also triggered by the observation that certain mistakes are made repeatedly by employees. Convincing others* – carried out by two persons in a leadership position and related to electronic documentation systems – were triggered by the perception that employees did not see the added value of the technology:

“I’m still in the process of convincing others during many shifts that this can be really helpful.” [P9].

A learning activity in the externalization category that was also reported by participants without leadership function was passing on suggestions to superiors*. This was triggered by the observation that some colleagues had difficulties in dealing with the electronic documentation system, caused by an increase in complexity. In this case, the learning activity was preceded by an elaboration learning activity, namely reflecting on how to make the system more manageable for these colleagues. Furthermore, passing on suggestions to superiors was triggered by a problem caused by the increased degree of formalisation. In addition, it was described that in the context of electronic documentation systems and information management systems, *information was shared* with colleagues. In one of these cases, the learning activity was preceded by looking up information in an online manual (expansion):

“And in the end, that wasn’t as hard as I thought it would be. But I could shine because nobody knew. I could say for the next three weeks, I know, ask me.” [P6].

Writing a standard operating procedure* together with colleagues in relation to electronic documentation systems was again mentioned by a participant in a leadership position. This was triggered in part by an anticipated knowledge gap of future colleagues. Related to this was determining the relevance of specific aspects* and formalizing them in the operating procedure. In this specific case, the concern that deviations in the timing of individual care steps in the documentation could lead to misinterpretation and be misused for control purposes triggered the learning activity:

“When I look at the planning of care measures and see an increased risk of decubitus, he must be positioned every three hours. And then I see that an employee only positions properly every second time in terms of time interval. And in my role as nurse manager, I can also call up everything from my laptop. So here I also see a great danger of misuse, for example, that I control my employees. […] Maybe there is a medication administration or a doctor’s visit and at the same time I should have positioned the patient. That’s why we don’t attach any importance to the time sequence of care in the documentation, and we have laid this down in the standard operating procedures from the outset.” [P5].

Table 5 contains an overview of all learning activities and their specific triggers due to the implementation of technology and related changes at work. It was developed iteratively and presents the analysed categories in context. It is based on the linked codes that refer to one incident. These cases were finally merged in the table.

**Table 5:** Learning activities due to the implementation of technology and related changes at work and their specific triggers

Learning activity	Trigger	Change in work characteristic			Technology	
***Elaboration***						
Experimenting	Usage of technology [P6, P9, P10]				Communication	Task-replacing
	Problem (due to increased degree of formalization) [P7]	Task			Communication	
Learning by doing	Usage of technology [P1, P2, P5, P10]				Communication	Task-replacing
Reflecting	Decreased temporal-spatial flexibility [P2]			Context	Communication	
	Increased complexity and related colleague behaviour [P2]	Task			Communication	
Testing something in a safe space	Usage of technology [P6]				Communication	
***Expansion***						
Asking colleagues	Problem [P1, P4, P10]				Communication	Task-replacing
	Problem (due to increased degree of formalization) [P7]	Task			Communication	
	Usage of technology [P2, P5, P7, P8]				Communication	Task-replacing
Asking experts	Problem [P1, P5]				Communication	
Asking multipliers	Problem (due to increased degree of formalization) [P6]	Task			Communication	
	Usage of technology [P3]				Communication	
Asking superiors	Problem [P4, P7]				Communication	Task-replacing
Looking up information	Problem [P1, P9, P10]				Communication	Task-replacing
	Knowledge gap (unusual task) [P2]				Communication	
	Knowledge gap (new task) [P6]	Task			Communication	
Participating in formal training	Usage of technology [P1, P2, P3, P4, P5, P6, P7, P9, P10]				Communication	Task-replacing
Reading the manual	Usage of technology [P10]					Task-replacing
Receiving advice from experts	Usage of technology [P5]				Communication	
Receiving briefing from colleagues	Usage of technology [P2]				Communication	
Receiving feedback from colleagues	Knowledge gap (new task) [P7]	Task				Task-replacing
Receive guidance from colleagues	Knowledge gap (new task) [P7]	Task				Task-replacing
***Externalization***						
Addressing common errors in team meetings	Colleague behaviour (repeated errors) [P3]				Communication	
Convincing others	Colleague behaviour (rejection of technology) [P3, P9]				Communication	
Determining the relevance of specific aspects	Perception of the risk of misuse (due to increased traceability) [P5]			Context	Communication	
Giving feedback	Change in responsibilities [P9]		Social		Communication	
	Colleague behaviour (repeated errors) [P3]				Communication	
Passing on suggestions to superiors	Increased complexity and related colleague behaviour [P2]	Task			Communication	
	Problem (due to increased degree of formalization) [P6]	Task			Communication	
Sharing information	Knowledge gap of colleagues (new task) [P6]	Task			Communication	
	Usage of technology [P1]				Communication	
Writing a standard operating procedure	Anticipated knowledge gap of colleagues (new task) [P5]	Task			Communication	

Note. *N* = 10, P = participant

## Discussion

The present study examined the changes at work caused by the implementation of technology and nurses’ professional development that arises as a result. The following section discusses the results in terms of the changes in work characteristics caused by the implementation of technology. Thereby, the differences between the technology categories are highlighted. Subsequently, the results on professional development induced by the implementation of a technology are discussed and hypotheses are derived from these findings.

### Changes in work characteristics due to the implementation of new technologies

Changes were reported for all technology categories and all categories of work characteristics. However, a look at the individual categories reveals differences: While the changes caused by the implementation of a communication supporting technology related to all categories (task, social, contextual), changes in certain work characteristics were mentioned for task-supporting and task-replacing technologies. The findings for communication supporting technologies align with earlier results, indicating that they can reduce strain (e.g., through automation and time savings) and enhance coordination of the care process (e.g., through direct, ward-independent access to patient data) [1]. Equally consistent with current literature are inconsistent results found (e.g., a decrease and increase in the time required for a task in relation to the same technology) as well as a decrease in autonomy (e.g., due to rigid documentation requirements, so that independent thinking and action is restricted) [1]. Regarding the degree of human involvement, it is notable that changes in social characteristics were only mentioned for communication supporting technologies. It remains unclear whether this circumstance can be explained by the fact that the function of these technologies is to connect people or whether there are other underlying reasons for this. However, it has already been shown and discussed that electronic documentation could replace personal exchanges between nursing staff [12]. Such change entails the risk of important information, such as insights based on intuition or professional experience, not being documented, and consequently not being communicated [1]. The study by Ihlebæk [33], for example, showed that the exclusive use of electronic documentation as a handover tool can lead to the loss of important information, such as information that is regarded as sensitive or unsuitable for recording. Furthermore, verbal exchange in the professional context is important to share informal knowledge, resolve uncertainties and provide support [33].

Changes were reported in relation to the implementation of task-supporting technologies. All findings for this technology category related to task characteristics and can be understood as a facilitation of work (e.g., the decrease in cognitive demands and the decrease in time required). These results align with conceptual considerations, as the main idea of these technologies is to support the execution of tasks [12] and thereby making them easier. Similarly, empirical evidence on how technologies in this category are changing work points to a facilitation of work, e.g., by reducing physical demands through patient lifts [34] or exoskeletons [13].

Changes regarding task-replacing technologies were mentioned in relation to task and context characteristics. There are also opposite findings, such as an increase and decrease in cognitive as well as emotional demands. These results are for example due to the facilitation of work tasks (e.g., through automation of task steps) while at the same time the systems are unfamiliar to use. These contradictory results are consistent with previous research, as the introduction of technology initially leads to a phase of adaptation in established work routines [1]. Moreover, it was reported that technologies in this category changed the content of the work tasks by taking over a specific task from the technology. This is of interest since the potential substitution of human labour with technology is being discussed in the nursing sector, too [35]. However, it is emphasized that high-quality care relies on the complex and situational decisions made by nursing staff, which cannot be replicated by technology [1]. In addition, taking on a physically and emotionally challenging task by a technology was perceived as positive in the present study, as it meant that more challenging tasks could be taken on, thereby enhancing the profession.

Further investigation of work characteristics in nursing and how it is changed by technology is needed. Especially since aspects such as physical and emotional exhaustion as well as depersonalization are positively related to turnover [36]. In contrast, it is negatively related to aspects such as satisfaction with the physical work environment, with the workplace, with the work content, with interpersonal relationships, as well as support from colleagues [36]. Therefore, more detailed research is needed into how technologies are changing work in nursing to address potentially negative effects. For example, the greater the perceived benefit of technology for the quality of care, the more positively nurses react to the resulting changes at work [37].

### Professional development arising from the implementation of new technologies

The following chapter illustrates the relationships between constructs. The basis for this was not provided by individual codes or cases, but by general observations across all data. References are therefore made in particular to Table 5. Overall, interview data indicates that the implementation of new technology in nursing leads to changes that are linked to professional development. In addition, the actual usage of technology and associated aspects such as problems and knowledge gaps trigger learning (see column “*Trigger*” in Table 5). This is conceptually plausible, since even without changes in work characteristics, newly implemented technologies are initially unfamiliar (e.g., in their handling and their integration into work processes) and thus affect work routines [e.g., 4, 5]. Looking at the links between the categories of technology and associated learning activities, contrasts emerge. First, it appears that the reported learning activities are related to communication supporting, and task-replacing technologies, while no learning activities were mentioned in relation to task-supporting technologies (see column “*Technology*” in Table 5). Regarding the latter, it was reported that nothing or little had to be learned to be able to use the technology. One explanation could be that these technologies (tablets with apps and label printers) are often already used in similar forms in both private and professional spheres, making knowledge transfer possible [e.g., 38]. In addition, it stands out that regarding task-supporting technologies, only changes at work were mentioned that indicate that work got easier, such as the automation of task steps, the decrease of cognitive demands and time savings (see column “*Task-supporting*” in Table 4). Even in the case of communication supporting and task-replacing technologies, changes in work characteristics that can be interpreted as a facilitation of work did not trigger learning in any case (see column “*Trigger*” in Table 5). On a conceptual basis, it could be argued that work that has become easier does not require direct adaptation and therefore learning does not occur. Based on these observations, the first two hypotheses can be derived:

#### Hypothesis 1

*Changes in work characteristics that lead to a disruption of work routines are positively related to professional development.*

#### Hypothesis 2

*Changes in work characteristics that make work easier are not related to professional development.*

The next level of consideration arises from the analysis of the individual components of professional development. Depending on the category, differences in triggers can be identified. On the one hand, elaboration resulted from changes in task characteristics and context characteristics due to the implementation of communication supporting technology (see column “*Trigger*” in section “*Elaboration*” in Table 5). On the other hand, it was triggered by the actual usage of communication supporting and task-replacing technology. This observation is consistent with the framework used, as this component of learning is primarily about using experience from and during work to develop one’s own competences [3].

Expansion was also triggered both by the usage of technology itself as well as by changes at work. However, when examining the specific triggers, it becomes clear that the learning activities related to a change were caused by associated problems or knowledge gaps due to new tasks (see column “*Trigger*” in section “*Expansion*” in Table 5). This observation is in line with the conceptual basis, as existing knowledge gaps and problems had to be solved by adding a new external input [3]. From this, the next hypothesis can be derived:

#### Hypothesis 3

**a**: *Facing problems due to the implementation of new technology is positively related to expansion.*

#### Hypothesis 3

**b**: *Facing knowledge gaps due to the implementation of new technology is positively related to expansion.*

Externalization was associated with changes in work characteristics from each category: For example, with changes in task content due to new tasks (task characteristic), changed responsibilities (social characteristics), or increased traceability (contextual characteristics). Moreover, externalization was triggered by social aspects, such as the behaviour of colleagues (see column “*Trigger*” in section “*Externalization*” in Table 5). In addition, these learning activities were in almost all instances carried out by persons in a leadership position [P3, P5, P9] or other staff responsibility (e.g., training of nursing students) [P2, P6].

#### Hypothesis 4

*Staff responsibility is positively related with externalisation*.

However, it remains unclear whether staff responsibility implies that nurses are more inclined to see this form of professional development as part of their role [39], or whether other factors are at play, such as a greater alertness to work-related changes and employees’ (learning) needs. Nevertheless, these results underline the relevance of nurse managers for learning at the workplace [40].

Further insights can be gained about the way learning takes place in the context of technology implementation. It becomes apparent that all mentioned learning activities of the category elaboration can be assigned to individual learning activities, whereas most of the learning activities of the category expansion and all learning activities of the category externalization can be assigned to social learning activities [28]. This shows the relevance of the social context for technology adoption processes [41] and workplace learning [42] in the nursing domain. It remains open whether there is a need to foster social learning opportunities in relation to elaboration [43] or whether it is more likely to take place in an individual setting.

Finally, it is important to note that communication supporting technologies, especially electronic documentation systems, were particularly mentioned in this study, both in inpatient and outpatient care. This could reflect the current prevalence of these technologies in the German nursing domain [35]. However, it also shows the importance of follow up studies that examine possible differences between the technologies and thus enable derivations for the different degrees of human involvement. Furthermore, more recent technologies, such as artificial intelligence, are largely still in the development phase in the nursing domain [44] and their future impact difficult to foresee.

It became visible that all three components of professional development are important in the adaptation process of nurses to the changed requirements of work due to the implementation of new technology. However, it also became apparent that especially regarding learning activities in the categories of expansion and externalisation, the social environment seems to play a key role in the nursing domain. It can be concluded from this that especially in the case of technologies where little prior knowledge is available and the existing knowledge base is not sufficient to solve problems autonomously, opportunities for social exchange among caregivers must be established. About externalisation, it becomes clear that especially nurses with staff responsibility, such as leadership position, share their knowledge and identify opportunities for improvement. However, this form of learning is not only important for the individual development of nurses, but also for the development of the team and the organisation. Therefore, in change management, attention should be paid to how this form of professional development can be promoted to nurses without extended responsibilities.

The results show how nurses adapt to the changed requirements of work due to the implementation of new technology, which work characteristics, learning activities and triggers are of importance and which differences exist regarding the three components of professional development. These findings can help to take specificities of the situation into account when planning implementation processes and to derive adapted requirements for the learning environment. Especially, as it is shown that the interaction between technology and social environment is of importance for the successful implementation of new technologies [45] and management strategies adapted to the situation and caregivers may be required during the change process [46].

### Limitations

Due to the exploratory nature of this study, with the actual research design, the results have limitations in terms of generalizability. This is also related to the small sample size and stem from a specific geographical area in Germany. The recruitment process might have caused that, participants having a positive attitude towards technology, felt more addressed by the call for study participation. However, the sample shows differences in terms of gender, age, and professional experience of the participants. Since the aim of this study was to gain in-depth insights into the relationships between new technologies, changes in work characteristics and professional development, limited generalizability and representativeness is not negatively affecting the quality of the results. In addition, the sample provided illustrative examples for all categories of professional development and all categories of technology. In line with the aim of this exploratory study, examples for the relationships between technologies and professional development were determined. The results provide the basis for hypotheses that can be tested in further studies with larger samples.

Another aspect that needs to be considered is that learning can be both explicit and implicit, and workplace learning in particular is often not recognised as such [47]. Therefore, it is possible that participants are not always aware of their learning activities or how they had learned something, and therefore could not report it in an interview. Furthermore, in relation to some learning activities, no explicit statements were made about their triggers. In these cases, it was assumed that the usage of the technology itself served as a trigger. Nevertheless, it is possible that specific triggers played a role in these cases, but that the participants were not aware of them themselves. Furthermore, from the fact that no learning activities were reported in the context of task-supporting technologies, cannot be derived that the adoption of these technologies will generally not result in any learning. Such outcomes may be due to common limitations of exploratory qualitative studies but can be used to emphasize the relevance of quantitative testing of the derived hypotheses. Especially, because, due to the exploratory nature of the present study, no statement can be made about the universality of the observed links, yet. Future studies should therefore systematically investigate the relationships between work characteristics and learning activities. Especially since different relationships in terms of the type of work characteristic (e.g., characteristics perceived as demands or resources) and the type of change (e.g., changes perceived as strains or reliefs as well as direction and intensity of the change) are to be expected [e.g., 48, 49].

### Implications for research

The aim of the present study was to investigate the relationships between technology, changes at work, the triggers for learning activities and the concrete learning activities that emerge from them. The results raised new questions and possibilities for future research. Firstly, the empirical testing of the derived hypotheses. The quantitative testing of the hypotheses with a large sample will provide a profound basis for practical implications. Therefore, future research should systematically analyse how changes in work characteristics due to the implementation of new technologies are related to professional development. Moreover, a targeted comparison of the three technology categories and related changes in work characteristics caused by their implementation could indicate different requirements in the implementation processes as well as differences in how to foster professional development. In this respect, it is also necessary to investigate which variables moderate the process of professional development triggered by new technologies, such as individual variables (e.g. motivation, attitude, age) or aspects of support (from colleagues and leaders) and the prevailing learning culture.

In addition, the group studied was relatively heterogeneous in terms of care sector and professional experience. A systematic comparison of different groups (e.g. outpatient vs. inpatient care, nurses with vs. without staff responsibility) or the targeted investigation of specific groups (e.g. intensive care, psychiatric care) could reveal differences in terms of technology and changes at work and thus uncover specific requirements for different fields of nursing. Furthermore, there is also a need for investigating the effects of intentional interventions (e.g., leadership, training) to foster professional development in situations of change at the workplace. Such insights would make it possible to design implementation processes constructively as well as specific interventions. To understand what role the implementation of technology plays in the development of nurses’ competences and how the learning activities are related to the different types of competences, future research should also investigate competences as a result of the learning activities. These findings can help to develop tailored implementation processes and derive adapted requirements for the learning environment, ensuring the successful integration of new technologies in nursing.

## Conclusion

This explorative study provides initial insights into the relationships between changes at work due to the implementation of new technologies and nurses’ professional development. The analysis of the interview data showed that the three categories of technology are changing work in nursing in different ways. It was found that, on the one hand, contradictory changes can occur regarding a certain technology and, on the other hand, that there are differences between the categories, for example regarding the facilitation of work. Learning activities from all three components of professional development emanate from these changes. Again, differences between the categories of technology were mentioned. Both the changes in work and related problems and knowledge gaps, as well as the usage of the technology itself were triggers for learning activities. The findings can be used by both nursing staff and their supervisors. On the one hand, the results make the various forms of professional development in the context of the implementation of new technology visible. This can raise nurses’ awareness of their own professional development by highlighting often unconscious, informal ways of learning. For example, it can be useful to know who in the team can be contacted if problems arise, where information can be found if there are knowledge gaps, or how information should be shared within the team so that everyone is informed. Furthermore, this study offers an opportunity to reflect on one’s own procedures and strategies. On the other hand, the results can help leaders to understand the development of their team members and create a suitable environment for this. It is of importance for leaders to know how their team members are developing in the context of change. From this, they can derive indications of how they can improve this, for instance by creating favourable conditions for social exchange or by managing reactions to change by convincing their team members. The results of the present study also suggest that nurses with staff responsibility themselves play a central role in the professional development of their employees and colleagues regarding externalization.

Compared to previous research, the present study provides initial insights into the links between new technology, changes at work, specific triggers for learning activities and the actual professional development. Although technology-related changes in nursing are currently a highly regarded research field, there research on how nurses respond to these changes in terms of professional development is missing. The present study therefore provides a first insight into the topic through concrete examples from practice. However, concrete implications for practice require subsequent research and empirical testing of the hypotheses.

## Electronic supplementary material

Below is the link to the electronic supplementary material.

Supplementary Material 1

## Data Availability

To protect the integrity, anonymity, and confidentiality of the participants and in accordance with the received ethics vote and General Data Protection Regulation (GDPR), data will not be shared.

## References

[1] Fachinger U, Mähs M. Digitalisierung und Pflege. In: Klauber J, Geraedts M, Friedrich J, Wasem J, editors. Krankenhaus-Report 2019. Berlin: Springer; 2019. pp. 115–28. 10.1007/978-3-662-58225-1_9.

[2] Skiba DJ. Nursing informatics education: from automation to connected care. Stud Health Technol Inf. 2017;232:9–19. 10.3233/978-1-61499-738-2-9. 28106577

[3] Simons PRJ, Ruijters MCP. Learning professionals: towards an integrated model. In: Boshuizen HPA, Bromme R, Gruber H, editors. Professional learning: gaps and transitions on the way from novice to expert. Innovation and change in professional education. Volume 2. Dordrecht: Springer; 2004. pp. 207–29. 10.1007/1-4020-2094-5_11.

[4] Bauer J, Gruber H. Workplace changes and workplace learning: advantages of an educational micro perspective. Int J Lifelong Educ. 2007;26(6):675–88. 10.1080/02601370701711364.

[5] Edmondson AC, Bohmer RM, Pisano GP. Disrupted routines: team learning and new technology implementation in hospitals. Adm Sci Q. 2001;46(4):685–716. 10.2307/3094828.

[6] Morgeson FP, Humphrey SE. Job and team design: toward a more integrative conceptualization of work design. In: Martocchio JJ, editor. Research in personnel and human resources management. Volume 27. Leeds: Emerald Group Publishing Limited; 2008. pp. 39–91. 10.1016/S0742-7301(08)27002-7.

[7] Moore EC, Tolley CL, Bates DW, Slight SP. A systematic review of the impact of health information technology on nurses’ time. J Am Med Inf Assn. 2020;27(5):798–807. 10.1093/jamia/ocz231. 10.1093/jamia/ocz231 PMC7309250 32159770

[8] Petrakaki D, Kornelakis A. We can only request what’s in our protocol’: technology and work autonomy in healthcare. New Technol Work Employ. 2016;31:223–37. 10.1111/ntwe.12072.

[9] Hong JY, Ivory CH, VanHouten CB, Simpson CL, Novak LL. Disappearing expertise in clinical automation: barcode medication administration and nurse autonomy. J Am Med Inf Assoc. 2021;28(2):232–8. 10.1093/jamia/ocaa135. 10.1093/jamia/ocaa135 PMC7883990 32909610

[10] Koltsida V, Jonasson LL. Registered nurses’ experiences of information technology use in home health care - from a sustainable development perspective. BMC Nurs. 2021;20:71. 10.1186/s12912-021-00583-6. 33933055 10.1186/s12912-021-00583-6 PMC8088614

[11] Chiang KF, Wang HH. Nurses’ experiences of using a smart mobile device application to assist home care for patients with chronic disease: a qualitative study. J Clin Nurs. 2016;25:2008–17. 10.1111/jocn.13231. 27136280 10.1111/jocn.13231

[12] Mulder RH, Beer P, Schierl K, Bornhaupt LR. Möglichkeiten für Professional Development durch die Veränderung von Arbeit als folge technologischer Entwicklungen im Gesundheitswesen. In: Weyland U, Reiber K, editors. Professionalisierung der Gesundheitsberufe. Beiheft 33 für die Zeitschrift für Berufs- und Wirtschaftspädagogik. Stuttgart: Franz Steiner; 2022. pp. 151–81. Available from: https://www.steiner-verlag.de/Professionalisierung-der-Gesundheitsberufe/9783515132886

[13] Vallée A. Exoskeleton technology in nursing practice: assessing effectiveness, usability, and impact on nurses’ quality of work life, a narrative review. BMC Nurs. 2024;23:156. 10.1186/s12912-024-01821-3. 38443892 10.1186/s12912-024-01821-3 PMC10913291

[14] Gough R, Ballardie R, Brewer P. New technology and nurses. Labour Ind. 2014;24:9–25. 10.1080/10301763.2013.877118.

[15] Krick T, Huter K, Domhoff D, Schmidt A, Rothgang H, Wolf-Ostermann K. Digital technology and nursing care: a scoping review on acceptance, effectiveness and efficiency studies of informal and formal care technologies. BMC Health Serv Res. 2019;19:400. 10.1186/s12913-019-4238-3. 31221133 10.1186/s12913-019-4238-3 PMC6585079

[16] McOmber JB. Technological autonomy and three definitions of technology. J Commun. 1999;49(3):137–53. 10.1111/j.1460-2466.1999.tb02809.x.

[17] Autor DH, Levy F, Murnane RJ. The skill content of recent technological change: an empirical exploration. Q J Econ. 2003;118(4):1279–333. 10.1162/003355303322552801.

[18] Piaget J. The development of thought. Equilibration of cognitive structures. New York: The Viking Penguin Inc; 1977.

[19] von Glasersfeld E. Piagets konstruktivistisches Modell: Wissen und Lernen. In: Rusch G, Schmidt G SJ, editors. Piaget und der radikale Konstruktivismus. Frankfurt am Main: Suhrkamp; 1994. pp. 16–42.

[20] Ellström PE. The many meanings of occupational competence and qualification. J Eur Ind Train. 1997;21(6/7):266–73. 10.1108/03090599710171567.

[21] Hirschmann K, Mulder RH. Effects of complexity of work tasks on informal learning at work in the IT domain. In: Messmann G, Segers M, Dochy F, editors. Informal learning at work: triggers, antecedents, and consequences. London: Routledge; 2018. pp. 40–62. 10.4324/9781315441962.

[22] Flanagan JC. The critical incident technique. Psychol Bull. 1954;51(4):327–58. 10.1037/h0061470. 13177800 10.1037/h0061470

[23] Schluter J, Seaton P, Chaboyer W. Critical incident technique: a user’s guide for nurse researchers. J Adv Nurs. 2008;61(1):107–14. 10.1111/j.1365-2648.2007.04490.x. 18173737 10.1111/j.1365-2648.2007.04490.x

[24] Mulder RH. Using critical incidents and vignette technique in HRD research to investigate learning activities and behaviour at work. In: Saunders MNK, Tosey P, editors. Handbook of research methods on human resource development. Cheltenham: Edward Elgar Publishing Limited; 2015. pp. 258–72. 10.4337/9781781009246.

[25] Schreier M. Qualitative content analysis. In: Flick U, editor. The SAGE handbook of qualitative data analysis. London: SAGE Publications Ltd; 2014. pp. 170–83. 10.4135/9781446282243.

[26] Beer P, Mulder RH. The effects of technological developments on work and their implications for continuous vocational education and training: a systematic review. Front Psychol. 2020;11:918. 10.3389/fpsyg.2020.00918. 32457688 10.3389/fpsyg.2020.00918 PMC7226038

[27] Koopmans H, Doornbos AJ, van Eekelen IM. Learning in interactive work situations: it takes two to Tango; why not invite both partners to dance? Hum Resour Dev Q. 2006;17(2):135–58. 10.1002/hrdq.1166.

[28] Mulder RH. Exploring feedback incidents, their characteristics and the informal learning activities that emanate from them. Eur J Train Dev. 2013;37(1):49–71. 10.1108/03090591311293284.

[29] Wood RE. Task complexity: definition of the construct. Organ Behav Hum Decis Process. 1986;37(1):60–82. 10.1016/0749-5978(86)90044-0.

[30] Campbell JL, Quincy C, Osserman J, Pedersen OK. Coding in-depth semistructured interviews: problems of unitization and intercoder reliability and agreement. Sociol Methods Res. 2013;42:294–320. 10.1177/0049124113500475.

[31] O’Connor C, Joffe H. Intercoder reliability in qualitative research: debates and practical guidelines. Int J Qual Methods. 2020;19. 10.1177/1609406919899220.

[32] Landis JR, Koch GG. The measurement of observer agreement for categorical data. Biometrics. 1977;33(1):159–74. 10.2307/2529310. 843571

[33] Ihlebæk HM. Lost in translation - Silent reporting and electronic patient records in nursing handovers: an ethnographic study. Int J Nurs Stud. 2020;109:103636. 10.1016/j.ijnurstu.2020.103636. 32593879 10.1016/j.ijnurstu.2020.103636

[34] Gold JE, Punnett L, Gore RJ, ProCare Research Team. Predictors of low back pain in nursing home workers after implementation of a safe resident handling programme. Occup Environ Med. 2017;74(6):389–95. 10.1136/oemed-2016-103930. 27919063 10.1136/oemed-2016-103930 PMC5860804

[35] Merda M, Schmidt K, Kähler B. Pflege 4.0– Einsatz moderner Technologien aus der Sicht professionell Pflegender. Forschungsbericht [Internet]. Hamburg: Berufsgenossenschaft für Gesundheitsdienst und Wohlfahrtspflege. 2017 Aug. Available from: https://www.bgw-online.de/DE/Medien-Service/Medien-Center/Medientypen/BGW-Broschueren/BGW09-14-002-Pflege-4-0-Einsatz-moderner-Technologien.html

[36] Bae SH. Comprehensive assessment of factors contributing to the actual turnover of newly licensed registered nurses working in acute care hospitals: a systematic review. BMC Nurs. 2023;22:31. 10.1186/s12912-023-01190-3. 36739408 10.1186/s12912-023-01190-3 PMC9899133

[37] Tsarfati B, Cojocaru D. Introducing computerized technology to nurses: a model based on cognitive instrumental and social influence processes. Healthcare. 2023;11(12):1788. 10.3390/healthcare11121788. 37372906 10.3390/healthcare11121788 PMC10298258

[38] Nokes TJ. Mechanisms of knowledge transfer. Think Reason. 2009;15(1):1–36. 10.1080/13546780802490186.

[39] Surakka T. The nurse Manager’s work in the hospital environment during the 1990s and 2000s: responsibility, accountability and expertise in nursing leadership. J Nurs Manage. 2008;16:525–34. 10.1111/j.1365-2834.2008.00901.x. 10.1111/j.1365-2834.2008.00901.x 18558923

[40] Yen M, Trede F, Patterson C. Learning in the workplace: the role of nurse managers. Aust Health Rev. 2016;40:286–91. 10.1071/AH15022. 26364126 10.1071/AH15022

[41] Coffetti E, Paans W, Krijnen WP, Roodbol PF, Finnema EJ, Zuidersma J. Measuring the relationship between reciprocity behaviour and technology readiness of nursing staff working in residential care and community nursing: a cross-sectional study. J Adv Nurs. 2024;00:1–12. 10.1111/jan.16337. 10.1111/jan.16337 PMC11810486 39031480

[42] Jantzen D. Refining nursing practice through workplace learning: a grounded theory. J Clin Nurs. 2019;28:2565–76. 10.1111/jocn.14841. 30807678 10.1111/jocn.14841

[43] Newton JM, Henderson A, Jolly B, Greaves J. A contemporary examination of workplace learning culture: an ethnomethodology study. Nurse Educ Today. 2015;35(1):91–6. 10.1016/j.nedt.2014.07.001. 25064265 10.1016/j.nedt.2014.07.001

[44] von Gerich H, Moen H, Block LJ, Chu CH, DeForest H, Hobensack M, et al. Artificial intelligence-based technologies in nursing: a scoping literature review of the evidence. Int J Nurs Stud. 2022;127:104153. 10.1016/j.ijnurstu.2021.104153. 35092870 10.1016/j.ijnurstu.2021.104153

[45] Chang F, Eriksson A, Östlund B. Discrepancies between expected and actual implementation: the process evaluation of PERS integration in nursing homes. Int J Environ Res Public Health. 2020;17(12):4245. 10.3390/ijerph17124245. 32545871 10.3390/ijerph17124245 PMC7344572

[46] Barchielli C, Marullo C, Bonciani M, Vainieri M. Nurses and the acceptance of innovations in technology-intensive contexts: the need for tailored management strategies. BMC Health Serv Res. 2021;21:639. 10.1186/s12913-021-06628-5. 34215228 10.1186/s12913-021-06628-5 PMC8253682

[47] Eraut M. Informal learning in the workplace. Stud Contin Educ. 2004;26(2):173–247. 10.1080/158037042000225245.

[48] van Ruysseveldt J, van Dijke M. When are workload and workplace learning opportunities related in a curvilinear manner? The moderating role of autonomy. J Vocat Behav. 2011;79:470–83. 10.1016/J.JVB.2011.03.003.

[49] Wielenga-Meijer EGA, Taris TW, Wigboldus DHJ, Kompier MAJ. Don’t bother me: learning as a function of task autonomy and cognitive demands. Hum Resour Dev Int. 2012;15(1):5–23. 10.1080/13678868.2011.646898.

