# Supplementary material for: New technologies and related changes at work as triggers for professional development in the nursing domain: an exploratory interview study
Source: BMC Nurs. 2025 Jun 17;24:657. doi: 10.1186/s12912-025-03291-7 (PMC12172304; doi:10.1186/s12912-025-03291-7)
Supplement: Supplementary file 1 — Supplementary Material 1 [file 12912_2025_3291_MOESM1_ESM.docx]

Additional file 1

| **Interview guide**  **– New technologies and related changes as triggers for professional development** | |
| --- | --- |
| First of all, thank you very much for taking some time for this interview.  It will take about 45 to 60 minutes and will focus on how you deal with new technologies in your day-to-day work. Are there any time restrictions that I should be aware of?  As mentioned in the information sheet, all data will be treated confidentially and only I will have access to this videorecording. Compliance with the General Data Protection Regulation and consideration of your rights are of the utmost importance to me.  With your consent, I will now start the videorecording. The interview will then be transcribed - personal details (such as places, names of colleagues, or your organization) will be anonymised.  By new technologies, I mean both devices (such as smartphones, blood glucose meters, etc.) and applications (such as mobile apps, documentation software, etc.). | *Introduction* |
| **Please think about the last time a new technology was introduced as part of your work and describe that situation.** | *Critical Incident* |
| Follow up questions |  |
| 1. What kind of technology was that?  - For which work activity / task do you use this technology? - What functions does the technology have that are relevant to your work? - How long ago was it introduced? | *Technology* |
| 1. How has this technology changed your work?  - To what extent have your work tasks changed? - To what extent have characteristics of your work changed? - What do you think about these changes? | *Changes at work* |
| 1. How did you deal with these changes?  - What did you have to acquire in this situation (for example knowledge, skills, or attitudes)? - How did you do this? | *Professional development / learning activities* |
| 1. What made you decide to do this? What caused your actions? | *Trigger* |
| 1. Is there anything else you would like to say or add? |  |
| Thank you for taking part in this interview. May I send you a questionnaire on your socio-demographic data by e-mail afterwards? | *Background variables*  *Ending* |
